# Hydrocarbonoclastic *Alcanivorax* Isolates Exhibit Different Physiological and Expression Responses to *n*-dodecane

**DOI:** 10.3389/fmicb.2016.02056

**Published:** 2016-12-21

**Authors:** Marta Barbato, Alberto Scoma, Francesca Mapelli, Rebecca De Smet, Ibrahim M. Banat, Daniele Daffonchio, Nico Boon, Sara Borin

**Affiliations:** ^1^Centre for Microbial Ecology and Technology, Ghent UniversityGhent, Belgium; ^2^Department of Food, Environmental and Nutritional Sciences, University of MilanMilan, Italy; ^3^Department of Medical and Forensic Pathology, University of GhentGhent, Belgium; ^4^School of Biomedical Sciences, University of UlsterColeraine, UK; ^5^Biological and Environmental Sciences and Engineering Division, King Abdullah University of Science and TechnologyThuwal, Saudi Arabia

**Keywords:** *Alcanivorax*, transcriptomics, alkanes, bioremediation, functional redundancy

## Abstract

Autochthonous microorganisms inhabiting hydrocarbon polluted marine environments play a fundamental role in natural attenuation and constitute promising resources for bioremediation approaches. *Alcanivorax* spp. members are ubiquitous in contaminated surface waters and are the first to flourish on a wide range of alkanes after an oil-spill. Following oil contamination, a transient community of different *Alcanivorax* spp. develop, but whether they use a similar physiological, cellular and transcriptomic response to hydrocarbon substrates is unknown. In order to identify which cellular mechanisms are implicated in alkane degradation, we investigated the response of two isolates belonging to different *Alcanivorax* species, *A. dieselolei* KS 293 and *A. borkumensis* SK2 growing on *n*-dodecane (C12) or on pyruvate. Both strains were equally able to grow on C12 but they activated different strategies to exploit it as carbon and energy source. The membrane morphology and hydrophobicity of SK2 changed remarkably, from neat and hydrophilic on pyruvate to indented and hydrophobic on C12, while no changes were observed in KS 293. In addition, SK2 accumulated a massive amount of intracellular grains when growing on pyruvate, which might constitute a carbon reservoir. Furthermore, SK2 significantly decreased medium surface tension with respect to KS 293 when growing on C12, as a putative result of higher production of biosurfactants. The transcriptomic responses of the two isolates were also highly different. KS 293 changes were relatively balanced when growing on C12 with respect to pyruvate, giving almost the same amount of upregulated (28%), downregulated (37%) and equally regulated (36%) genes, while SK2 transcription was upregulated for most of the genes (81%) when growing on pyruvate when compared to C12. While both strains, having similar genomic background in genes related to hydrocarbon metabolism, retained the same capability to grow on C12, they nevertheless presented very different physiological, cellular and transcriptomic landscapes.

## Introduction

Removal of petroleum-hydrocarbons in marine environments by means of mechanical methods is used as a primary response during oil spill, targeting a large part of the recoverable oil from water. Nonetheless, the total and ultimate oil removal completely relies on the process of natural attenuation carried on by autochthonous microbial organisms inhabiting the affected areas. Among the many bacterial phylogenetic groups able to degrade hydrocarbon pollutants in marine environments, *Alcanivorax* represents a key player. This genus consists of Gram-negative, aerobic, halophilic γ-proteobacteria and its representatives are ubiquitous: they have been detected in a wide geographical area from the North Sea to the Pacific Ocean, in both surface waters and deep-sea sediments (Head et al., 2006). Although they represent a very low proportion of the total bacterial community of pristine waters, these microbes are the first to bloom after superficial oil spills, reaching 80–90% of the whole bacterial community (Syutsubo et al., 2001). Due to their hydrocarbonoclastic nature they can utilize only few organic acids (e.g., acetate and pyruvate; Naether et al., 2013) but are able to use as carbon and energy source a wide range of alkanes. This preference toward alkane molecules consumption makes *Alcanivorax* genus one of the most promising targets for the setup of bioaugmentation-based remediation biotechnologies.

The *Alcanivorax* genus was firstly described in 1998 by Yakimov et al., who isolated a hydrocarbon degrading bacterium capable of using aliphatic hydrocarbons as the sole source of carbon and energy from waters and sediments collected near the Borkum Island, in the North Sea (Yakimov et al., 1998). Since then, *A. borkumensis* became one of the most investigated hydrocarbon degrading bacterial species, often used as a reference strain in studies involving hydrocarbon degradation in marine environments (Sabirova et al., 2008, 2006; Naether et al., 2013; Hassanshahian et al., 2014; Bookstaver et al., 2015). The *Alcanivorax* genus presently comprises 11 species, of which *A. dieselolei* (Liu and Shao, 2005) represents one of the most environmentally spread. *A. dieselolei* was firstly isolated in 2001 from Bohai Sea surface waters and deep-sea sediments of the Pacific Ocean (Liu and Shao, 2005), and it has been detected in a wide range of marine costal and pelagic environments (Wang et al., 2010; Kostka et al., 2011), tropical and temperate areas (Wang et al., 2010; Kostka et al., 2011; Hassanshahian et al., 2012), in association with other marine organisms (Campos et al., 2015), as well as in hydrocarbon-contaminated saline soils (Dastgheib et al., 2011). The distribution of this specie in the environment mirrors its physiological traits, which allow its representatives to adapt to a wide range of different environmental conditions.

Both *A. dieselolei* and *A. borkumensis* show the peculiar traits of hydrocarbonoclastic bacteria. They can degrade a wide range of hydrocarbons: *A*. *dieselolei* can degrade a wide spectrum of linear and branched alkanes (Liu and Shao, 2005; Wang and Shao, 2014), while *A. borkumensis* can degrade linear alkanes, cycloalkanes and isoprenoids (Yakimov et al., 1998). Many genes encoding for enzymes initiating the degradation of hydrocarbons have been detected in the genomes of the two species: *alk*B and p450 are present in both, while *alm*A and *dad*B are peculiar of *A. dieselolei* (Sabirova et al., 2006, 2011; Wang et al., 2010; Liu et al., 2011; Wang and Shao, 2012, 2014; Li and Shao, 2014). As many other hydrocarbonoclastic bacteria, they are able to produce biosurfactants to facilitate their access to alkanes. *A. dieselolei* produces a proline lipid active in a wide range of pH and temperature (Qiao and Shao, 2010), while *A. borkumensis* produces many different glycolipid biosurfactants (Abraham et al., 1998; Yakimov et al., 1998). Furthermore, since it has been proved the absence of the *Alcanivorax* genus in deep sea environments contaminated by hydrocarbons (Hazen et al., 2010; Bælum et al., 2012), the capability of this genus to adapt and survive at high hydrostatic pressure has been recently investigated in *A. borkumensis* SK2 and *A. dieselolei* KS 293, highlighting different strategies to cope with this environmental stressor (Scoma et al., 2016a,b). The growth of both strains was significantly impaired already at 5 and 10 mPa, but while in KS 293 respiration and cell integrity were not affected by mild hydrostatic pressure, they were severely impaired in SK2. At the same time, only SK2 was able to activate the production of ectoine as a resistance mechanisms against hydrostatic pressure (Scoma et al., 2016a,b). Finally, in the presence of hydrocarbons *A. borkumensis* is also able to modify its membrane hydrophobicity (HC) and to synthesize intracellular reservoirs of carbon (Naether et al., 2013).

These two species share some fundamental functions for HC degradation and bioremediation of polluted environments. Functional redundancy is a key feature in environmental microbial assemblages, and the presence of strains having same function but different transcriptomic and physiological adaptations has been proposed to provide better community services (Wittebolle et al., 2009). The effectiveness of autochthonous or bioaugmented hydrocarbonoclastic bacterial communities in marine oil spill remediation is therefore depending on the coexistence of strains showing highly efficient hydrocarbon utilization and exploiting different metabolisms and pathway regulation. Not much is known about whether or not different adaptation strategies to oil exist within the many species belonging to this hydrocarbonoclastic genus. With this aim, in the present work we investigated the physiological, cellular and transcriptomic response of two hydrocarbonoclastic isolates with similar genetic background, namely *A. borkumensis* SK2 (SK2) and *A. dieselolei* KS 293 (KS 293). Strains were grown with either *n*-dodecane (C12) or pyruvate as unique carbon source, with a view to establish whether similar or uniform approaches are adopted in response to hydrocarbon exposure and whether this could affect their individual role in the process of hydrocarbon pollution remediation.

## Materials and methods

### Strains and growth conditions

*Alcanivorax dieselolei* KS 293 (Barbato et al., 2015) was isolated from surface waters collected from the Levantine basin of the Mediterranean Sea (Mapelli et al., 2013) after enrichment with ONR7a mineral growth medium and diesel oil 1% (v/v) as the sole carbon source. *Alcanivorax borkumensis* SK2 type strain was chosen as a control strain. Strains were grown axenically in 250 mL glass bottles containing 100 mL of ONR7a media supplied with either 1% *n*-dodecane (C12) (v/v) or pyruvate (PYR) (w/v) as the sole carbon and energy source, at room temperature, for 7 days without mixing. All *Alcanivorax* cultures were inoculated at an optical density (OD) at 610 nm equal to 0.05. Experiments were conducted in eight independent replicates. In an additional experiment, *Alcanivorax* cells of either SK2 or KS 293 were grown to the late exponential phase on either pyruvate or C12, collected by centrifugation and re-suspended in three independent replicates in a carbon-free ONR7a medium to test their capacity to grow with no added carbon source.

### Microbial analyses

Cell growth was assessed through flow cytometry (FC) analyses. Flow cytometry was carried out according to De Roy et al. (2012). Utilization of the supplied carbon sources was indirectly evaluated by measuring pH variation in the growth media at the end of the incubation time, provided that either C12 or pyruvate were supplied as sole carbon source. pH values were determined with a 744 pH meter (Herisau, Metrohm, Switzerland). Cell hydrophobicity was evaluated through the Microbial Adhesion To Hydrocarbon (MATH) test, modified after Rosenberg et al. (1980). Cells were pelleted at 1699 g for 10 min and rinsed twice in PUM buffer (22.2 g/L K_2_HPO_4_.3H_2_O; 7.26 g/L KH_2_PO_4_; 1.8 g/L UREA; 0.2 g/L MgSO_4_.7H_2_O; pH was adjusted to 7.1). Finally, cells were resuspended in PUM buffer to a final volume of 1.2 mL and OD_610_ value of about 1. After measuring the rinsed-cells OD_610_ (OD_i_), cells were supplied with 0.1 mL *n*-hexadecane (C16) and incubated at 28°C for 10 min. Afterwards, cells were mixed for 2 min and left to rest for 15 min. Finally, the OD_610_ of the aqueous phase was measured again (OD_f_). The relative amount of cells bound to *n*-hexadecane was calculated as follows:

(1) $$\begin{array}{l} \left({\left({\text{OD}}_{\text{i}}-{\text{OD}}_{\text{f}}\right)}^{*}100\right)/{\text{OD}}_{\text{i}} \end{array}$$

### Chemical and microscopy analyses

The extraction and quantification of phospholipid fatty acids (PLFAs) were performed following the method described by Denef et al. (2007). Total lipids were extracted from the cell pellet using phosphate buffer/chloroform/methanol at a 0.9:1:2 ratios and partitioned into neutral, glyco- and phospho-lipids by solid phase extraction. Phospholipids were methylated by mild alkaline methanolysis (using methanolic KOH) to form fatty acid methyl esters (FAMEs), which were analyzed by capillary gas chromatography-combustion-isotope ratio mass spectrometry (GC-c-IRMS) (GC-C/Delta PLUS XP Thermo Scientific) *via* a GC/C III interface as described by Denef et al. (2007). Standard fatty acid nomenclature was used to describe PLFAs. For each sample, the abundance of individual PLFAs was calculated in absolute amounts of C and then converted to mole percentage PLFA-C.

#### Surface tension

Surface tension measurements were carried out on the culture supernatant after 7 days of incubation using the Du Nouy method (Marchant and Banat, 2014) which uses a digital tensiometer (K12, KRÜSS GmbH, Hamburg) equipped with a platinum-iridium ring. Distilled water was used to calibrate the instrument, with the surface tension for pure water equal to 72.8 mN/m at 20°C.

#### Transmission electron microscopy (TEM)

Cells were collected by centrifugation at 1699 g for 10 min at 4°C (Sorval RC5c PLUS, Beckman, Suarlée, Belgium). The supernatant was discarded and the pellet fixed for 48 h in 0.1 M cacodylate buffer containing 4% paraformaldehyde and 5% glutaraldehyde. After subsequent fixation in 1% osmium tetroxide for 3 h, samples were dehydrated in a series of alcohol treatments (15 min in 50% alcohol, 15 min in 70% alcohol, 15 min in 90% alcohol, 3x 30 min in 100% alcohol) and embedded in Epon medium (Aurion, Wageningen, The Netherlands). Ultra-thin sections of 60 nm were cut and contrasted with uranyl acetate and lead nitrate. Samples were finally imaged with a Zeiss TEM900 transmission electron microscopy (Carl Zeiss, Oberkochen, Germany) at 50 kV.

#### Genome comparison

KS 293 (Barbato et al., 2015) and SK2 genomes (Golyshin et al., 2003) have been compared through the RAST (Rapid Annotation using Subsystem Technology) platform (Overbeek et al., 2014) according to the authors instructions.

#### Organic acid analyses

Volatile fatty acids between C2 and C8 (including isoforms C4–C6) were measured by gas chromatography (GC-2014, Shimadzu®, The Netherlands) with DB-FFAP 123-3232 column (30 m × 0.32 mm × 0.25 μm; Agilent, Belgium) and a flame ionization detector (FID). Liquid samples were conditioned with sulfuric acid and sodium chloride and 2-methyl hexanoic acid as internal standard for quantification of further extraction with diethyl ether. Prepared sample (1 μL) was injected at 200°C with a split ratio of 60 and a purge flow of 3 mL min^−1^. Oven temperature increased by 6°C min^−1^ from 110 to 165°C where it was kept for 2 min. FID had a temperature of 220°C. The carrier gas was N_2_ at a flow rate of 2.49 mL min^−1^. Lactate, formate, pyruvate, glycerol, 1,2 propanediol, 1,3 propanediol, methanol and ethanol were analyzed by ion chromatography (930 Compact IC Flex, Metrohm, Switzerland) with inline bicarbonate removal (Metrohm CO_2_ suppressor). Separation was done on a Metrosep organic acids (250/7.8) column at 35°C behind a Metrosep organic acids (4.6) guard column. The eluent was 1 mM H_2_SO_4_ and the flow rate 0.5 mL min^−1^. An 850 IC conductivity detector was used for detection of eluted components. Detection was enhanced using a chemical suppression module to replace protons with Li-cations (Metrohm suppressor module with 0.5 M LiCl). The sample aspiration needle was cleaned with acetone between each analysis. The lower limit of quantification was 1 mg L^−1^.

#### C12 analyses

Total residual C12 concentration at the end of the growth was evaluated by adding *n*-hexane (C6) (Sigma Aldrich, Belgium) to the cultures in a ratio 1:4 (v:v), respectively. Cultures were thoroughly shaken for 1 min and set aside for 15 min. The upper layer constituted of C6 and extracted C12 was sampled and injected in a GC as reported in Scoma et al. (2016b).

### Transcriptomic analysis

Cells were collected at the stationary phase of growth. Three independently grown cultures were pooled together to average the cells response to the carbon source. Pooled cultures were centrifuged at 4°C for 5 min 17,949 g (Sorval RC5c PLUS, Beckman, Suarlée, Belgium) and the pellet immediately stored at −80°C. Later on, pellets were thawed and RNA quickly extracted.

#### RNA extraction and QC

RNA was isolated using the Rneasy Mini Kit (Qiagen, Antwerp, Belgium) following the manufacturer instructions. On-column DNase digestion was performed during the RNA extraction. The RNA concentration was determined using NanoDrop 2000 UV-Vis spectrophotometer (Thermo Scientific, Waltham, MA, USA). Samples of SK2 growing with pyruvate or C12 had a 661.6 and 356.4 ng/μL yield, respectively, while KS 293 had a 266.5 and 126.4 ng/μL yield, respectively. RNA quality control was performed using the 2100 Bioanalyzer microfluidic gel electrophoresis system (Agilent Technologies, Santa Clara, USA).

#### RNA library prep and sequencing

Libraries for RNA-sequencing were prepared using the ScriptSeq Complete (Bacteria) sample prep kit (Epicentre—Illumina, San Diego, CA, USA). Starting material (1000 ng) of total RNA was depleted of rRNAs using Ribo-Zero magnetic bead based capture-probe system (Illumina, Hayward, USA). Remaining RNA (including mRNAs, lin-cRNAs and other RNA species) was subsequently purified (Agencourt RNA- Clean XP, Beckman Coulter, Brea, CA, USA) and fragmented using enzymatic fragmentation. First strand synthesis and second strand synthesis were performed and double stranded cDNA was purified (AgencourtAMPure XP, Beckman Coulter). RNA stranded libraries were pre-amplified and purified (AgencourtAMPure XP, Beckman Coulter). Library size distribution was validated and quality inspected using the 2100 Bioanalyzer (high sensitivity DNA chip, Agilent Technologies). High quality libraries were quantified using the Qubit Fluorometer (Life Technologies, Carlsbad, CA, USA), concentration normalized and samples pooled according to number of reads. Sequencing was performed on a NextSeq500 instrument using Mid Output sequencing kit (150 cycles) according to manufacturer's instructions (Illumina, Hayward, USA).

#### Data processing workflow

Data analysis pipeline was based on the Tuxedo software package (Oracle, Redwood Shores, CA, USA). Components of the RNA-seq analysis pipeline included Bowtie2 (v. 2.2.2), TopHat (v2.0.11) and Cufflinks (v2.2.1). TopHat is a fast splice junction mapper for RNA-Seq reads, which aligns sequencing reads to the reference genome using the sequence aligner Bowtie2. TopHat uses sequence alignments to identify splice junctions for both known and novel transcripts. Cufflinks takes the alignment results from TopHat to assemble the aligned sequences into transcripts, constructing a map of the transcriptome. A previously reported transcript annotation was used to guide the assembly process for *A. borkumensis* SK2 (Schneiker et al., 2006) and *A. dieselolei* KS 293 (Liu and Shao, 2005).

#### Data analysis

Genes were grouped according to orthologous clusters using the database provided by Ortholuge DB (Whiteside et al., 2013). Only clusters classified as supporting-species-divergence (SSD) were considered for further analyses and the rest were discarded (Borderline-SSD, Divergent-SSD, Similar Non-SSD and unevaluated orthologs [RBB]). Up and downregulation analysis were expressed on a log2 basis, indicating fold changes in fragments per kilobase of transcript per million mapped reads (FPKM) between the same strain (SK2 or KS 293) supplied with C12 or pyruvate and between the different strains, but growing with the same carbon source. Gene clusters were arbitrarily considered upregulated when their log2 fold change was higher than 0.5. On the contrary, it was considered that downregulated genes had a −0.5 fold change. All gene clusters that were expressed between −0.5 and 0.5 were considered to be unaffected by a change in carbon source or between strains. The ±0.5 log2 fold change was established in order to have a reasonable compromise in the definition of both upregulated and unaffected genes, provided that a higher threshold may be more appropriate to assess upregulation but would result into an overestimation of unaffected genes. Nonetheless, we considered strongly upregulated those genes with log_2_FC higher than 2 and strongly downregulated those with log_2_FC lower than −2. Final analysis of up and down-regulated genes, was carried out using the database provided by the Kyoto Encyclopaedia of Genes and Genomes (KEGG) (www.genome.jp/kegg/).

### Statistical analysis

Results were expressed as mean values of experiments made in 3–8 independent replicates. Bars in the graphs indicate a 95% confidence interval (95% CI) calculated using a Student *t*-test with a two-sided distribution. Statistical significance was assessed using a non-parametric test (Mann-Whitney test) which considered a two-sided distribution with 95% CI.

## Results

### *A. dieselolei* KS 293 and *A. borkumensis* SK2 genome comparison features

KS 293 (Barbato et al., 2015) and SK2 genomes (Golyshin et al., 2003) have been compared through the RAST platform (Overbeek et al., 2014). KS 293 showed larger genome and higher %GC and coding sequence number respect to SK2 (Table 1). Despite these differences, the two genomes shared a functional identity of 80.3%, equivalent to 1952 functional gene categories having the same function over a total of 2413. 340 gene categories were peculiar of KS 293 while only 139 of SK2, but none of them was specifically correlated to alkane metabolism (Table S1). In particular, the amino acid sequence identity of genes directly involved in the first step of alkane sensing and degradation was analog in both strains (Table S2).

**Table 1:** **General genomic features of ***A. dieselolei*** KS 293 and ***A. borkumensis*** SK2**.

	***A. dieselolei*** **KS 293**	***A. borkumensis*** **SK2**
Genome size (bp)	4,790,658	3,120,143
GC content (%)	61.4	54.7
Contigs	57	1
Coding sequence	4445	2916
Number of subsystem	459	430
Number of RNA	47	51
Isolation source	Eastern Mediterranean Sea	North Sea

### Growth capacity in *A. dieselolei* KS 293 and *A. borkumensis* SK2 on different carbon sources

**Figure 1:**
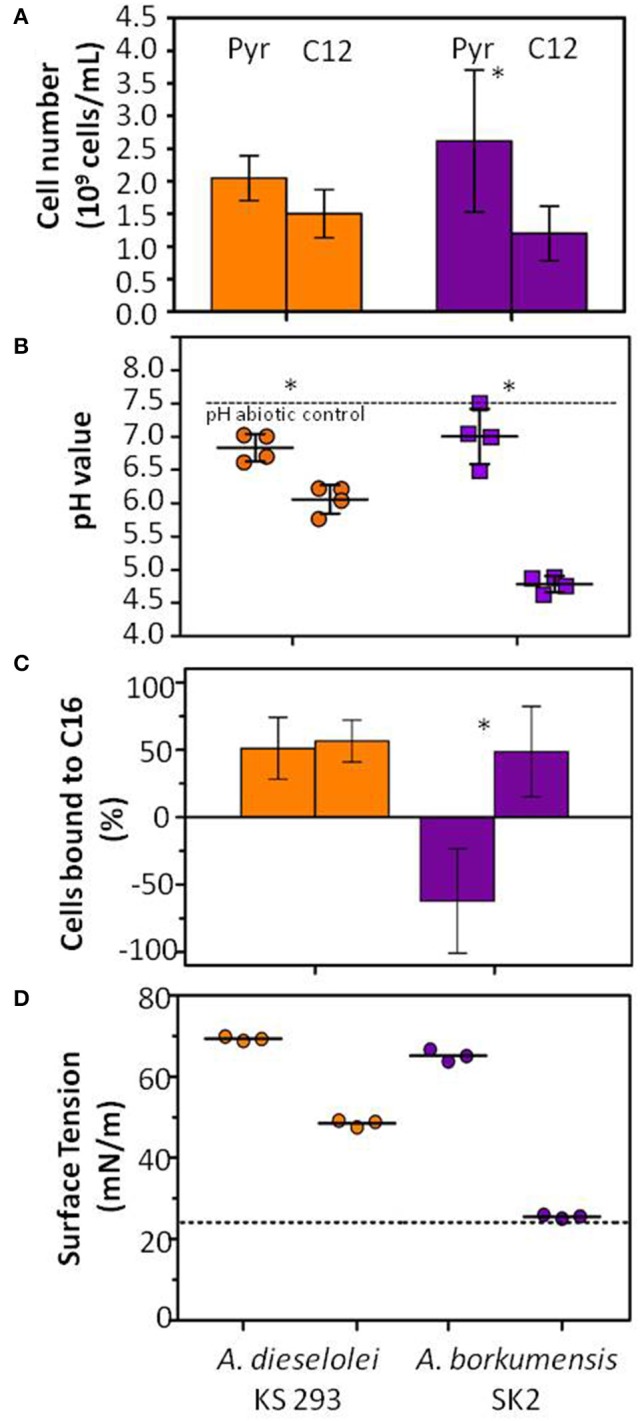
**Growth evaluation and physiological features of ***A. borkumensis*** SK2 and ***A. dieselolei*** KS 293 strains after 7 days incubation on pyruvate (PYR) or ***n***-dodecane (C12)**. Bars indicate the 95% confidence interval (CI). Stars indicate significantly different values (*p* < 0.05). **(A)** Total cell number evaluation with flow cytometry. **(B)** Measurement of pH variation of the culture media after incubation. **(C)** MATH test, percentage of cells bound to *n*-exadecane (C16). **(D)** Surface tension of the culture media.

Both strains were able to grow with either C12 or pyruvate as sole carbon source (Figure 1A). The total cell number at the stationary phase, after 7 days of incubation, was assessed through flow cytometry. All the conditions achieved statistically similar final cell numbers (*p* > 0.05), with the exception of strain SK2 supplied with pyruvate (Figure 1A), which showed higher growth capacity when growing on this carbon source (*p* < 0.05).

The initial pH of the media (7.5) remained constant throughout the experiment in abiotic controls, while it decreased in all inoculated samples (Figure 1B): in both strains pH values significantly decreased when C12 was supplied (*p* < 0.05). In particular, pH was significantly lower in SK2 growing on C12 as compared to pyruvate (*p* < 0.05).

Final consumption capacity of either pyruvate or C12 was assessed by evaluating the grams of carbon per liter of culture (g_carbon_ L^−1^) at the end of the incubation time (Table S3). The results showed no statistical difference (*p* > 0.05) neither between strains growing on the same carbon source, nor when comparing the same strain with either carbon source.

At the end of the incubation time, also the production of organic acids was evaluated. Neither strains produced these compounds when growing on C12 (detection limit 1 mg L^−1^), but they produced lactate, formate and acetate after growth on pyruvate (Table S4).

### Cell membrane properties

**Figure 2:**
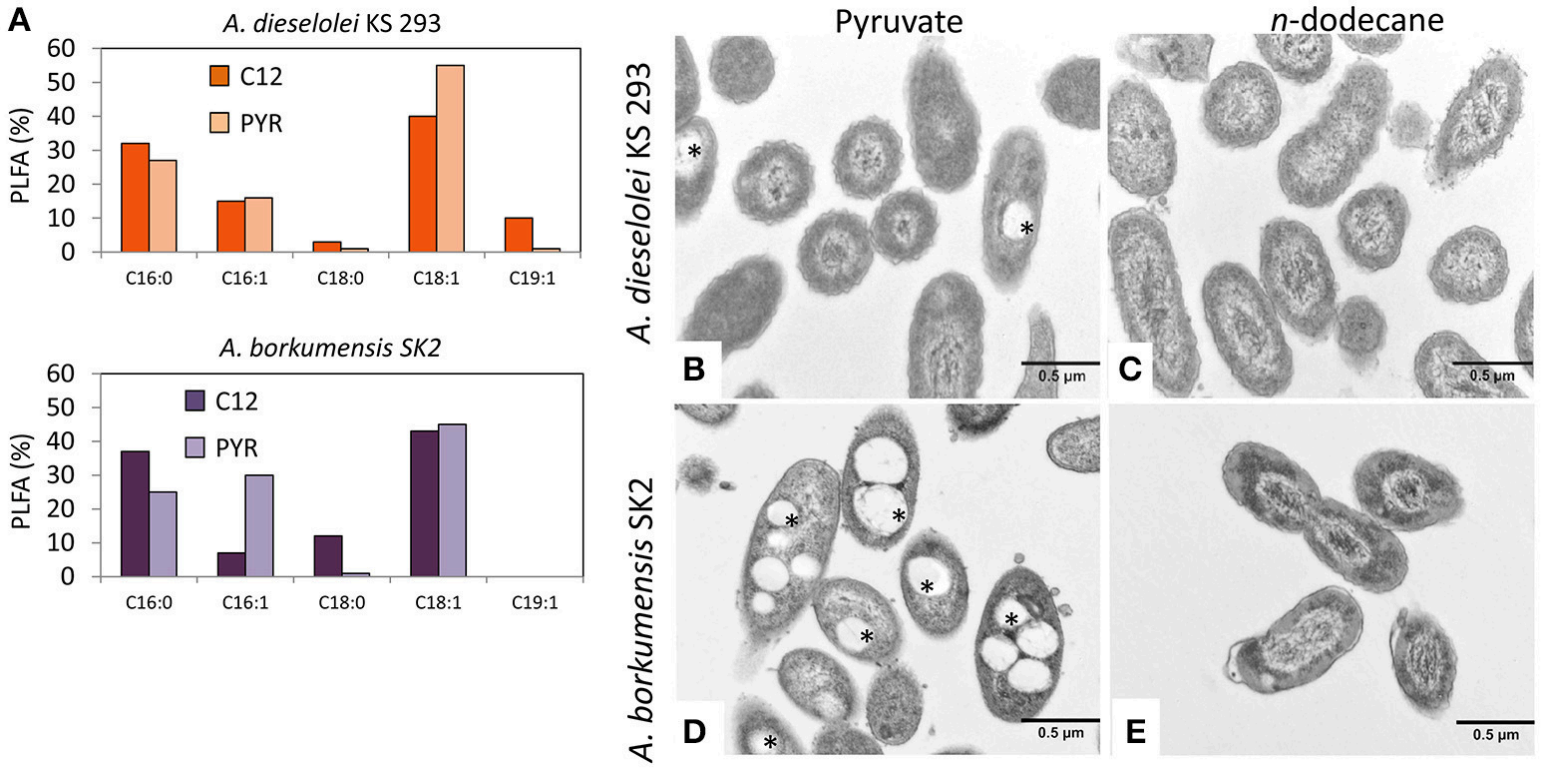
**(A)** Relative abundance of PLFA in cell membranes after 7 days incubation on pyruvate (PYR) or *n*-dodecane (C12) in *A. dieselolei* KS 293 and *A. borkumensis* SK2. **(B–E)** Transmission electron microscopy of cells of *A. dieselolei* KS 293 **(B,C)** and *A. borkumensis* SK2 **(D,E)** after 7 days of growth on pyruvate (PYR) **(B,D)** or *n*-dodecane (C12) **(C,E)** as the sole carbon source. Stars indicate electron transparent intracellular bodies inside the cells.

Hydrophobicity tests performed on cell cultures grown on either pyruvate or C12 indicated no significant changes in *A. dieselolei* KS 293 cells (*p* > 0.05), which were always highly hydrophobic. Conversely, *A. borkumensis* SK2 cells were highly hydrophilic when grown on pyruvate and switched to a hydrophobic behavior on C12 (Figure 1C). These results were further investigated by assessing the capacity of the cells to carry over C12. Cells grown on C12 and subsequently re-inoculated in mineral medium with no carbon source showed growth both in the case of SK2 (2 generations) and even more in KS 293 (5 generations), suggesting that a fraction of the C12 previously supplied remained stored intracellularly or was externally attached to the cells due to their superficial hydrophobicity. No growth was observed with cells previously grown on pyruvate when re-suspended in a carbon-free medium, despite accumulation of intracellular reservoirs (Figures 2B,D). The surface tension of the culture medium showed lower values for both strains growing on C12, possibly due to the production of biosurfactant molecules. The decrease was particularly significant in SK2 where such value reached the maximum reduction possible to surface tension in the water-based broth medium equal to ≈27 mN/m, while in KS 293 it reached a value of ≈48 mN/m (Figure 1D).

The impact of different carbon sources on membrane PLFA profiles (C11–C30) was investigated. The two strains showed minor differences in PLFA profiles. In both bacteria, the total amount of unsaturated, rather than saturated PLFAs was higher with the two carbon sources; however, we observed a slight increase of saturated PLFA in both bacteria when growing with C12 if compared to pyruvate (Figure 2A).

Also, ultra-thin sections of SK2 and KS 293 cells grown on C12 and pyruvate revealed significant differences in membrane morphology (Figures 2B–E). Cell membranes in both the strains appeared rather indented (Figures 2B,C,E), while only SK2 grown with pyruvate showed neat outer profile (Figure 2D). Provided that in this condition SK2 showed highly hydrophilic cells with the lowest capacity to adhere to hydrocarbons (Figure 1C), the neat membrane morphology could be related to cell surface hydrophilicity. Furthermore, the two strains showed electron-transparent inclusion bodies attributable to carbon reservoirs only when growing on pyruvate (Figures 2B,D), showing a massive accumulation in SK2.

### Transcriptomic analysis

In each dataset a total of 2307 gene clusters were identified. Their annotation contained 1487 sequences belonging to the SSD ortholog class, which were further associated to different functional pathways in the KEGG database. Of the remaining gene clusters, 47, 57, and 1 sequences were classified as borderline-SSD, divergent non-SSD and similar non-SSD, respectively, with 715 gene clusters not classified.

#### General gene expression level

**Figure 3:**
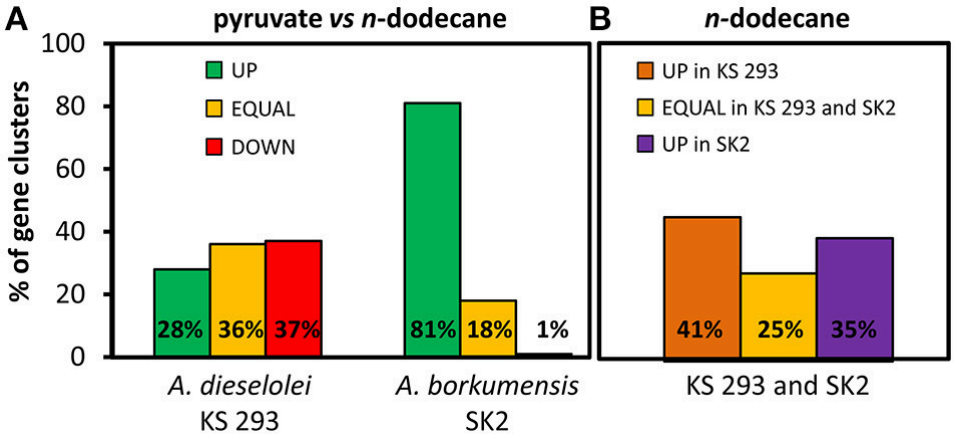
**General transcriptomic features. (A)** Percentage of gene clusters up-, down- or equally regulated in *A. dieselolei* KS 293 and *A. borkumensis* SK2 growing with pyruvate compared to *n*-dodecane. **(B)** Comparison of gene clusters transcription (%) in *A. dieselolei* KS 293 and *A. borkumensis* SK2 when growing with *n*-dodecane.

Gene expression levels differed markedly among samples (Figure 3A). Gene expression in SK2 was remarkably impacted by the carbon source, with 81% of genes upregulated when growing on pyruvate as compared to C12, contrary to the less affected response observed in KS 293 (28, 36, and 37% respectively up-, unaffected and downregulated genes). Notably, in SK2 only 1% of the genes were upregulated when growing on C12 as compared with pyruvate, and they were not related to alkane metabolism or to specific metabolic pathways. SK2 overall metabolism appeared to be upregulated in the presence of pyruvate, indicating a rampant gene transcription with this substrate rather than with C12 (Tables S2, S5–S8). Comparing the two strains growing on C12, they showed different transcription profiles with only 25% of the genes equally regulated (Figure 3B). 41% of genes were upregulated in KS 293, while 35% of genes were upregulated in SK2.

#### Genes involved in hydrocarbon metabolism

##### Alkane uptake and metabolism

**Figure 4:**
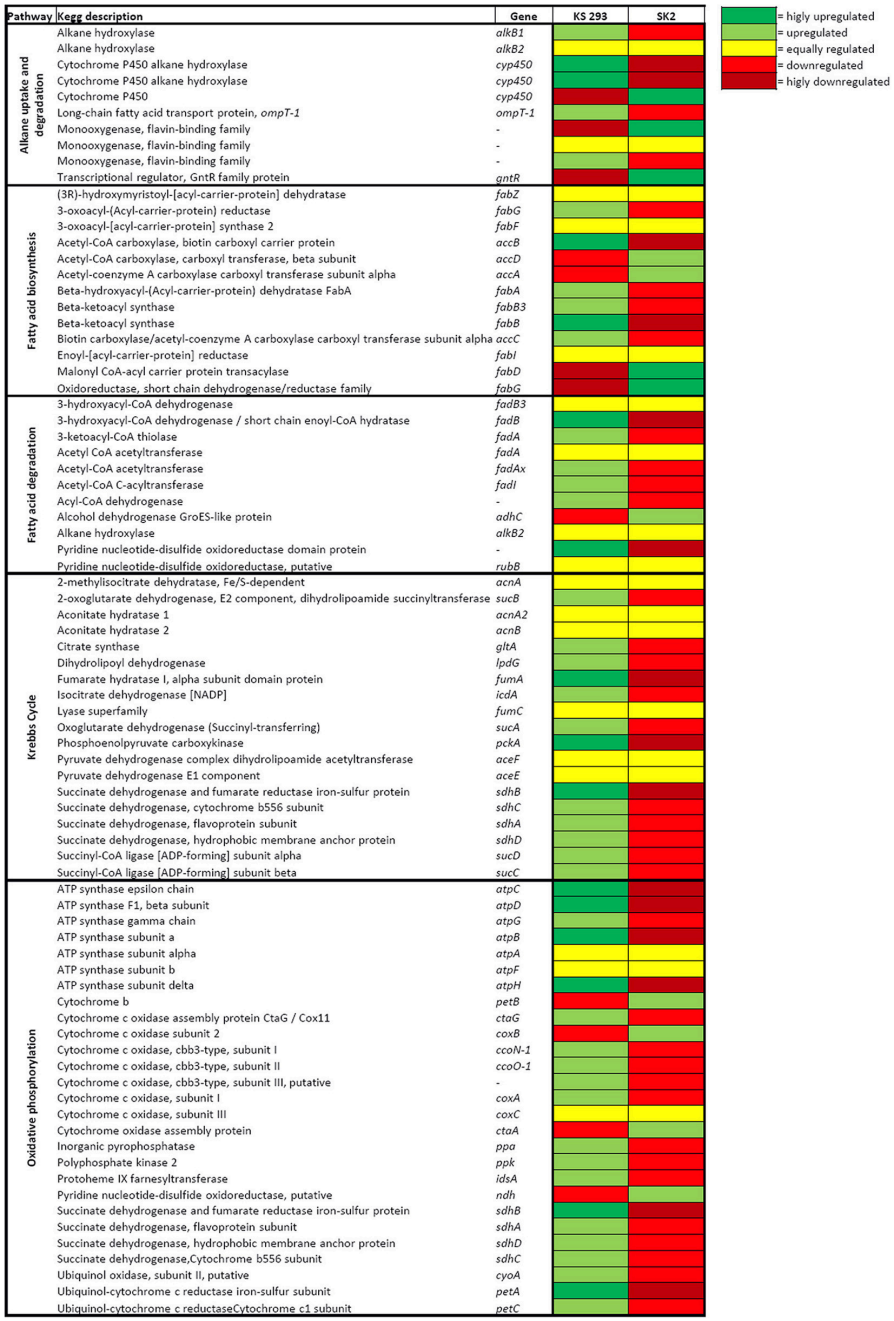
**Heat map of the transcription level of genes involved in hydrocarbon degradation and metabolism**. Yellow indicates the same level of transcription, light green indicates upregulation, dark green indicates strong upregulation, red indicates downregulation and dark red indicates strong downregulation.

Genes encoding for several known enzymes involved in hydrocarbon degradation, such as cytochrome P450 alkane hydroxylases, alkane 1-monooxygenases and monooxygenases flavin-binding family proteins were detected in the genome of both strains, as well as their transcriptional regulators and genes involved in the uptake of alkanes (Figure 4; Table S2). During growth on C12, expression of 3 out of 7 genes involved in the first step of alkane degradation was higher in KS 293 with respect to SK2 (B5T_02075, B5T_00721, and B5T_02052). The transcriptional regulator of the GntR family (ABO_0121 in SK2 and B5T_00102 in KS 293) was, nevertheless, strongly downregulated in KS 293 as compared to SK2, although the same level of expression was observed for the adjacent-located genes alkane monooxygenase *alkB2* (ABO_0122 and B5T_00103). As compared to SK2, KS 293 upregulated genes involved in alkane sensing and assimilation, i.e., *ompT* (B5T_02055; ABO_0193), encoding for a long-chain fatty acid transport protein involved in the alkane assimilation and *ompS* (B5T_01485; ABO_1688), which is responsible for initiating the signaling cascade for the alkane detection. Transcription level of these genes was coherent with the substrate utilized only in KS 293, i.e., upregulated when growing on C12 as compared with pyruvate.

##### Fatty acid metabolism, Krebs cycle and oxidative phosphorylation

In hydrocarbonoclastic bacteria, alkane degradation directly leads to the synthesis of fatty acids (Rojo, 2009). As the latter can encounter different metabolic routes, the transcription level of genes involved in both the biosynthesis and degradation of fatty acids was compared in KS 293 and SK2 growing with C12 (Figure 4; Table S2). In KS 293 a general upregulation of the genes involved in fatty acid β-oxidation was observed, potentially leading to a higher production of acetyl-CoA. Consistently, in this strain a higher transcription of almost all the genes involved in the Krebs cycle and in oxidative phosphorylation was detected (Figure 4; Table S2), despite their transcription level was higher when KS 293 was growing with pyruvate. In particular, KS 293 cells showed a strong upregulation of *sdhB* (B5T_02684, log_2_FC = 2.46) encoding for the succinate dehydrogenase/ fumarate reductase, directly involved in both the Krebs cycle and oxidative phosphorylation, and of the genes encoding for the ATP synthase complex. On the contrary, in SK2 we observed a strong upregulation of the *fabD* transcription (ABO_1068, log_2_FC = 2.60) (Figure 4; Table S2). This gene encodes for an S-maloniltransferase, which is essential for the initiation of the fatty acid biosynthesis in bacteria.

#### Genes involved in biosurfactant production and secretion

**Figure 5:**
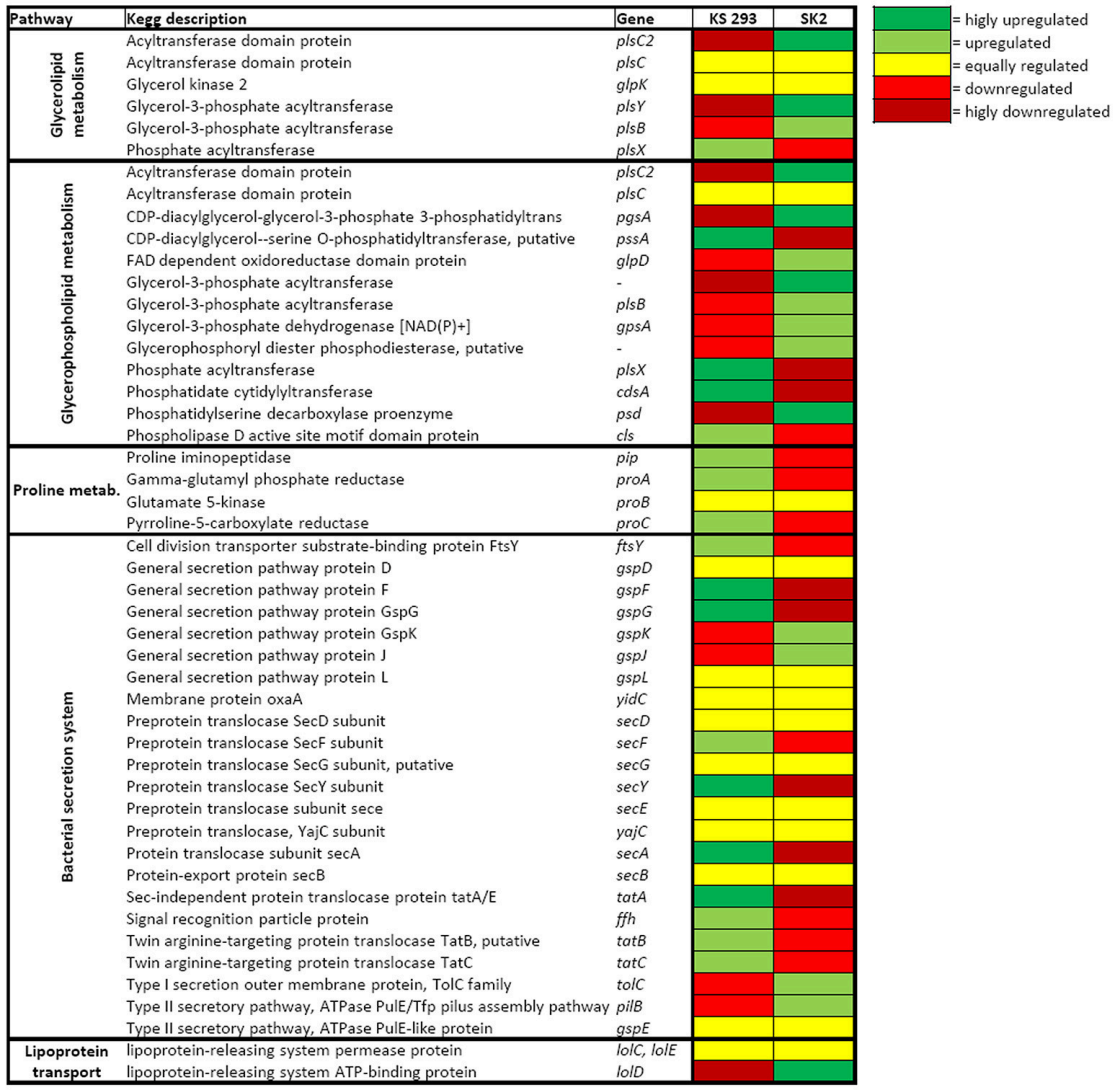
**Heat map of the transcription level of genes involved in biosurfactant production and secretion**. Yellow indicates the same level of transcription, light green indicates upregulation, dark green indicates strong upregulation, red indicates downregulation and dark red indicates strong downregulation.

Both isolates showed the expression of genes related to biosurfactant production when growing on C12, but preferred different classes of molecules. Strain SK2 growing with C12 upregulated, with respect to KS 293, most of the genes involved in glycerolipid and glycerophospholipid metabolism: *pls* genes encoding for acetyltransferase enzymes (ABO_2058, *plsY*, log_2_FC = 4.68 and ABO_0683, *plsC2*, log_2_FC = 3.29) and the gene *psd* (ABO_2213, log_2_FC = 3.32) encoding for a phosphatidylserine decarboxylase that catalyzes the release of a CO_2_ molecules from phosphatidylserine. In addition, SK2 strongly upregulated the *lolD* gene (ABO_1050, log_2_FC = 2.41) involved in the lipoprotein-releasing system (Figure 5, Table S5). On the contrary, in KS 293 almost all genes involved in proline synthesis were upregulated together with the gene *pssA* (B5T_03693, log_2_FC = 3.37) encoding for an enzyme directly involved in the synthesis of phosphatidylserine.

Genes for biosurfactant secretion were also differently expressed in the two strains. When growing with C12, SK2 upregulated genes encoding for the Type I secretion system as compared to KS 293, while KS 293 upregulated genes for Sec system and Twin arginine translocation system (Figure 5, Table S5). Nonetheless, 42 of the 48 detected genes involved in biosurfactant production and transport were downregulated in SK2 when supplying C12 as the sole carbon source, as compared to pyruvate (Table S5). The opposite is true for KS 293, in which 18 genes were upregulated and only 7 were downregulated in the presence of C12 rather than pyruvate. In addition, proline metabolism was equally regulated with both carbon sources in KS 293 (Table S5).

#### Genes involved in cellular wall and membrane biosynthesis

Biosynthesis of peptidoglycans was similarly regulated in KS 293 and SK2 when growing with C12 (Figure S1; Table S6). Only few genes followed a peculiar activation pattern: *mur*B gene involved in biosynthesis of murein monomers (ABO_1059, log_2_FC = 4.03) was upregulated in SK2 and the genes *ddl*, responsible for the synthesis of D-ala/D-ala dymer (B5T_03486, log_2_FC = 2.16), and *murD*, responsible for the addition of the second amino acid of the peptide chain (B5T_03490, log_2_FC = 2.67) were upregulated in KS 293. On the contrary, the transcription level of the genes involved in lipopolysaccharide biosynthesis revealed an upregulation of 3 out of 6 genes directly involved in the biosynthesis of the core region in SK2 (*waaA* ABO_2501, log_2_FC = 2.45; *waaF* ABO_2300, log_2_FC = 1.15; *waaP3* ABO_22296, log_2_FC = 1.78). Lipid A related genes and genes involved in the transport of the lipopolysaccharide across the cellular membrane were equally transcribed in the two strains (Figure S1, Table S6). Supply of C12 in KS 293 cells slightly upregulated genes involved in peptidoglycan final assemblage and in lipopolysaccharide biosynthesis with respect to pyruvate (Table S6).

Despite differences in PLFA profiles (Figure 2A), transcription of the genes involved in the biosynthesis of unsaturated fatty acids showed minor differences between SK2 and KS 293 growing on C12. Only the gene *yciA* (B5T_04371, log_2_FC = 3.99), involved in the final step of the synthesis of palmitic acid (C_16:0_), was strongly upregulated in KS 293 with respect to SK2 (Figure S1, Table S6), while it was equally transcribed when KS 293 grew using pyruvate.

#### Genes involved in synthesis of intracellular reservoirs of carbon

**Figure 6:**
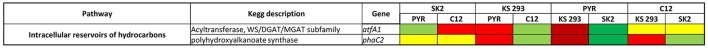
**Heat map of the transcription level of genes involved in intracellular reservoirs of carbon synthesis**. Yellow indicates the same level of transcription, light green indicates upregulation, dark green indicates strong upregulation, red indicates downregulation and dark red indicates strong downregulation.

In both the strains we identified the presence of two genes directly involved in the synthesis of carbon reservoirs as intracellular grains: *phaC2* and *atfA1* (Figure 6, Table S7). *phaC2* (ABO_1418; B5T_ 02346) is involved in the butanoate metabolism, and encodes for a polyhydroxyalcanoate synthase usually responsible for the synthesis of poly-β-hydroxybutyrate. Transcription levels of *phaC2* did not change in SK2 with either C12 or pyruvate, but were higher if compared with KS 293. *atfA1* (ABO_2742; B5T_04434) gene encodes for an acetyltransferase involved in the synthesis of triacylglycerols (TAGs) and wax esters (WEs) as storage lipids. Its transcription was upregulated in SK2 cells growing on pyruvate as compared to C12 (log_2_FC = 1.66). High expression levels of this gene were unique to SK2 growing on pyruvate, and resulted strongly downregulated in KS 293 growing on both pyruvate and C12. This observation is consistent with the observation of numerous intracellular bodies in SK2 cells supplied with pyruvate (Figures 2B–E).

#### Genes involved in ion uptake and chemiotaxis

When growing on C12, most of the genes involved in nitrogen and phosphorous assimilation were upregulated in KS 293 with respect to SK2 (Figure S2, Table S8). Particularly, a strong upregulation of the genes encoding for the ABC-transporter and phosphotransferase system specific for nitrate uptake (nasD, B5T_01565, log_2_FC = 2.24; ptsN, B5T_03579, log_2_FC = 2.45) and of one gene encoding for one subunit of the phosphate ABC transporter was observed (pstS, B5t_05368, log_2_FC = 2.12). In KS 293 growing on C12 the genes involved in nitrate, Fe(III) and iron complex uptake were upregulated as compared to pyruvate, while genes responsible for uptake of phosphorous were downregulated (Figure S2, Table S8). In SK2, as for most of the transcription profile, all the genes involved in ion uptake were downregulated when growing with C12 when compared to pyruvate.

A differential regulation of the genes involved in chemotaxis was observed in the two strains growing on C12 (Figure S2, Table S8). The transcription level of the chemotaxis regulatory subunits *cheR* and *cheB* (ABO_0105 and ABO_1310, respectively) was higher in SK2, while the genes encoding for the pili response regulator PilG and for the TonB dependent receptor domain protein OmpS (B5T_00068 and B5T_01485) were transcribed at a higher level in KS 293. Moreover, a comparison between the transcription level of genes in KS 293 growing with C12 and pyruvate showed that those encoding for OmpS, CheR, and CheB were upregulated in the presence of C12, while *pilG* was highly transcribed in the presence of pyruvate (Table S8). Almost all genes involved in chemotaxis were downregulated in SK2 growing with C12 as compared to pyruvate, in agreement with the general downregulation of the whole gene transcription profile. The only exception was represented by *ompS*, whose transcription level did not differ with either carbon source, suggesting a significant role of this gene in SK2.

## Discussion

High amount of hydrocarbons is not a widespread feature of seas and oceans (Halpern et al., 2008) and petroleum release may cause significant harm to marine life (Paul et al., 2013). Microbial degradation represents the ultimate process for the complete clean-up of oil polluted sites, and relays mainly on hydrocarbonoclastic bacteria, such as *Alcanivorax* spp. which commonly blooms after superficial oil spills (Hara et al., 2004) and whose peculiar metabolism allows them to use almost exclusively hydrocarbons for their growth. In the present study, the direct comparison of two different hydrocarbonoclastic *Alcanivorax* strains aimed at understanding their functional redundancy in terms of common and exclusive responses to alkanes. The two strains did not only shared this peculiar metabolism, but were also phylogenetically related and displayed a quite high functional genome identity. The activation of different metabolic strategies in specific nutritional scenarios, i.e., growing on C12 or on pyruvate, was investigated with a cross-disciplinary approach using molecular and physiological tools.

SK2 showed a very strong overexpression of most of the genes when growing with pyruvate rather than C12 (Figure 3A), including genes directly involved in alkane degradation such as *alkB* and *cyp450* (Table S2). However, the gene expression level of SK2 was comparable with KS 293 when both the strains were supplied with C12. Pyruvate in SK2 triggered a generalized high level of gene transcription, a phenomenon that has not been described in previous works on SK2 transcriptomics, in which the growth on pyruvate was compared with the growth on different alkanes provided as the sole carbon sources (Sabirova et al., 2011; Naether et al., 2013). This response may correlate with the observed significantly higher final cell number obtained when growing on this carbon source (Figure 1A), along with the shift to (i) surface hydrophilicity (Figure 1C), (ii) neat membrane profile (Figure 2D), and (iii) massive accumulation of intracellular bodies (Figure 2D) and certainly requires a deeper insight into SK2 metabolism and adaptation mechanisms to environmental changes. On the contrary, C12 triggered alternative but complementary adaptation mechanisms in SK2, which differed from KS 293 response.

### Hydrocarbon bioavailability, uptake, and metabolism

The hydrophobic nature of hydrocarbons limits their water solubility and, therefore, bacterial access to oil. One of the most common strategies to counteract this problem is to increase hydrocarbon availability by producing biosurfactants, molecules composed of both lipophilic and hydrophilic moieties (Banat et al., 2010). Both *A. dieselolei* and *A. borkumensis* are described in the literature to produce biosurfactant molecules in order to increase the bioavailability of lipophilic substrates (Marchant and Banat, 2012). In *A. borkumensis* SK2 many different glycolipid biosurfactants have been characterized, and their most common structure is an anionic glucose lipid composed by a glucose molecule connected to tetrameric β-hydroxy-decanoic acid (Abraham et al., 1998; Yakimov et al., 1998). The only characterized biosurfactant produced by *A. dieselolei* strain B5, is a lipopeptide composed by a proline residue bound to three different fatty acids (C_14:0_, C_16:0_, and C_18:0_) (Qiao and Shao, 2010). In the present work, reduced surface tension in cells growing on C12 in both strains confirmed the production of surface-active molecules, although in SK2 a more pronounced effect was assessed (Figure 1D). Decrease in surface tension during SK2 growth on C12 is coherent to the enhanced expression level of genes involved in glycerolipid and glycerophospholipid metabolism (Figure 5, Table S5). On the contrary, KS 293 upregulated the genes involved in proline metabolism (Figure 5, Table S5) together with the gene *yciA* (Figure S1, Table S6), responsible for the synthesis of the unsaturated fatty acid C_16:0_, confirming the production of a specific proline lipid biosurfactant which synthesis was observed exclusively in the *A. dieselolei* species (Qiao and Shao, 2010).

A key feature of hydrocarbonoclastic bacteria is their membrane composition. Depending on its hydrophobicity, the cell membrane may become more lipophilic when hydrocarbons are present, thus enhancing their capability to adhere to hydrophobic compounds (Ron and Rosenberg, 2002; Wick et al., 2002; de Carvalho et al., 2009). In accordance with previous reports (Naether et al., 2013), SK2 surface hydrophobicity was increased when growing on C12 as compared to pyruvate, inducing a net shift in cell capacity to adhere to *n*-hexadecane, and a change in membrane morphology from neat to indented (Figures 2B–E). An indented cell membrane could be related to a higher capability to adhere to hydrophobic substances, which would be an advantageous trait for hydrocarbonoclastic bacteria. In the present study, this was reflected in the capacity to store C12 molecules by the cell, allowing degradation at a later stage when cells are moved in a carbon-free medium. On the contrary, KS 293 did not show morpho-physiological adaptations induced by the supplied carbon source, as growth on pyruvate and C12 yielded comparable membrane hydrophobicity, cell adherence to hydrocarbons and membrane morphology.

Linear alkanes are lipophilic substances that, due to their nature, can easily insert through cell membrane double phospholipid layer, potentially resulting in cellular death by lysis (Heipieper and Martinez, 2010). Both KS 293 and SK2 showed similar PLFA response to C12, with a slightly increasing saturated fatty acid relative abundance (Figure 2A). This may reflect an adaptation strategy to C12 as an attempt to reduce cell membrane fluidity, thus preventing the uncontrolled entrance of the linear alkane. The similar physiologic and morphologic cellular membrane features of the two strains growing with C12 are indicative of analogous adaptation strategies of the membrane toward the presence of hydrocarbons, as also confirmed by the comparable transcription of the genes involved in the biosynthesis of cellular membrane constituents (Figure S1, Table S6).

Hydrocarbons are processed and degraded inside the cells, nonetheless the mechanism by which linear alkanes cross the cellular membrane is still unclear, and in recent years the involvement of transmembrane transporters has been suggested (Sabirova et al., 2011; Wang and Shao, 2014). *A. dieselolei* B5 possesses transporter proteins specific for alkane of different lengths: OmpT-1, OmpT-2, and OmpT-3, respectively allowing long-, medium- and short-chain alkanes to enter the cells (Wang and Shao, 2014). While genes encoding for OmpT-2 and OmpT-3 could not be detected in KS 293 transcriptome, the expression of the *ompT-1* gene in cells grown on C12 showed higher values than SK2 (Figure 4, Table S2). Wang and Shao (2014) suggested that OmpT-1 alone is not sufficient to sustain *A. dieselolei* B5 growth in the presence of C12 and, although Sabirova et al. (2011) proposed the outer membrane lipoprotein Blc to be involved in alkane uptake in SK2, the corresponding gene was not detected in the present transcriptomic analyses. The mechanisms responsible for short-medium length alkane transport in *Alcanivorax* representatives need therefore further consideration.

Once entering the cell, a three-step activation process initiates hydrocarbon degradation, where alkanes are first converted into alcohols, then to aldehydes and finally to fatty acids (Rojo, 2009). The first reaction is carried out by alkane hydroxylases enzymes (mainly AlkB and Cyp450) specific for alkanes of different length (Syutsubo et al., 2001; Hara et al., 2004; Rojo, 2009; Wang and Shao, 2014). This enzymatic apparatus has been thoroughly investigated in the last years, and its action mechanism uncovered in *A. dieselolei, A. borkumensis* and many other hydrocarbon degraders (Van Beilen et al., 2003; Rojo, 2009; Liu et al., 2011). KS 293 and SK2 genomes harbor both *alkB* and *cyp450* genes, but they were differentially transcribed and regulated (Figure 4, Table S2).

The subsequent conversion of alcohol to aldehyde relies on a dehydrogenase (*adhC*) whose transcription was upregulated in SK2 with respect to KS 293 (Figure 4, Table S2). The aldehyde is then converted to fatty acid, which can be degraded to acetyl-CoA through β-oxidation, constitute cellular membranes and exopolymeric substances, or be converted into storage materials (Rojo, 2009; Naether et al., 2013). Expression level of β-oxidation related genes in SK2 was equal or reduced as compared to KS 293. KS 293 also showed higher transcription levels for most of the fatty acid biosynthesis related genes and for the genes involved in Krebs cycle and oxidative phosphorylation (Figure 4, Table S2). Therefore, the main destiny of fatty acids directly deriving from C12 utilization in SK2 appears to be the formation of membrane structures or specific metabolites such as biosurfactant molecules, while in KS 293 it appears to be the production of ATP. Overall, genes involved in C12 mineralization and CO_2_ production were transcribed at higher levels in KS 293 than in SK2, in contrast to (1) the much lower pH value of SK2 cell supernatant at the end of the incubation time with C12 (Figure 1B) and (2) the statistical similarity between the carbon consumption capacity (g_carbon_ L^−1^; Table S3). We analyzed the amount of fermentation products accumulated by the cultures at the end of the incubation to test whether other factors could have accounted for the pH change. While cultures grown on C12 did not produce any organic acid (detection limit 1 mg L^−1^), they released traces of lactate, formate and acetate when grown on pyruvate (Table S4). The pKa of these organic acids is very low as compared to ONR7a medium initial pH (3.86, 3.77, and 4.76 *vs*. 7.5, respectively). However, cultures grown on pyruvate were statistically more alkaline than those grown on C12 (*p* < 0.05), indicating that the strong decrease in pH with either carbon source was due to other factors. In light of the transcriptomic results, the observed pH changes in SK2 growth media may be dependent on extracellular molecules, with biosurfactants a possible candidate.

Fatty acids resulting from hydrocarbon metabolism can also be stored as carbon reservoirs in intracellular bodies. To build intracellular reservoirs of carbon is a common strategy among bacteria when the environment has an unbalanced C:N ratio, i.e., when the reduced N supply is perceived as limiting for protein synthesis and cell replication (Zinn et al., 2001), a condition demonstrated in KS 293 with a high transcription level of *ntrB* that acts as sensor for low nitrogen availability (Figure S2, Table S8). The oligotrophic marine environments not chronically polluted by hydrocarbons offer limited opportunities to hydrocarbonoclastic bacteria with a narrow metabolic range apart from hydrocarbons (Yakimov et al., 1998; Liu and Shao, 2005), and the capability to store carbon reservoirs intracellularly could significantly increase their chances to survive. Accumulation of hexadecane-derived wax esters (WEs) has been indeed observed in *Marinobacter hydrocarbonoclasticus* and *A. borkumensis* (Kalscheuer et al., 2007; Grossi et al., 2010). In our experimental setting, a rather abundant carbon load was supplied to the strains in order to simulate culture conditions resulting from a spill (Sabirova et al., 2006). The *phaC2* gene involved in the synthesis of poly-β-hydroxybutyrate (PHB) was upregulated in KS 293, when growing on C12 respect to pyruvate, and not affected by carbon source in SK2. However, in both strains no PHB accumulation was detected, as previously revealed for *A. borkumensis* (Sabirova et al., 2006; Kalscheuer et al., 2007; Manilla-Pérez et al., 2010). The gene *atfA1* was suggested to encode for an enzyme involved in the synthesis of triacyl glycerols (TAGs) and WEs in *A. borkumensis* (Kalscheuer et al., 2007). Transcription levels of these genes were indeed much higher in SK2 growing on pyruvate rather than C12 (Figure 6), in agreement with an overabundant accumulation of electron-transparent intracellular grains in these cells (Figure 2D). Previous studies on *A. borkumensis* indicated the existence of alternative, uncharacterized metabolic routes for TAGs and WEs production, as intracellular grains of neutral lipids were observed notwithstanding a complete *atfA1* knock out (Kalscheuer et al., 2007). These findings may account for the small but detectable electron-transparent grains accumulated in KS 293 grown on pyruvate (Figure 2B), in which transcription levels of both *phaC2* and *atfA1* were particularly low (Figure 6). The expression level of this gene was, nevertheless, comparable in both KS 293 and SK2 when growing on C12, and the lack of inclusion bodies when growing on this substrate suggests that this alkane may not trigger intracellular carbon accumulation. Moreover, the positive transcriptional response of PHB synthesis related genes was not followed by their actual accumulation in the cell, nor it was for TAGs or WEs, whose target gene expression level was unaffected, indicating that excess carbon might not be a sufficient condition to trigger the synthesis of intracellular storage of carbon.

Chemiotaxis has been recognized as an advantage for hydrocarbonoclastic bacteria such as *A. dieselolei* B5 to detect the entrance of petroleum-hydrocarbons in the cell environment (Wang and Shao, 2014). Genes related to this function (*ompS*, Wang and Shao, 2014) are present in the genome of both strains, and when growing on C12 they were transcribed at significantly higher levels in KS 293 (Figure S2, Table S8). This suggests that other functions not directly linked to alkane metabolism could diversify the response of different hydrocarbonoclastic bacteria to hydrocarbon substrates.

Taken together, these data bring evidence of many common and some exclusive responses to oil in hydrocarbonoclastic isolates harboring the same genomic background. When using an alkane as carbon and energy source, the two strains showed comparable growth capacity and surface hydrophobicity as a strategy to increase the interaction with the lipophilic substrate. Furthermore, they shared the same cellular membrane composition and physiological properties toward C12 utilization, with similar pathways of alkane activation, and both overexpressed genes related to biosurfactant production as a strategy to increase alkane availability. These evidences confirm that the two strains share the same functions in terms of metabolic pathways sustaining the capacity to grow on alkanes. Nonetheless, their transcription patterns showed functional redundancy in key metabolic pathways, as differential responses in the genes expressed for alkane activation and regulation and for the production of surfactant molecules (glycolipids in SK2, lipopeptides in KS 293). The most remarkable difference between the strains resided in their adaptation mode to the different substrates. SK2 had significantly higher efficiency to grow on substrates alternative to alkane (pyruvate) and changed many key physiological aspects when growing on C12: the outer membrane changed from neat to indented and became highly hydrophobic to better interact with the lipophilic substrate; moreover, a significant decrease in surface tension of the cell supernatant was assessed, putatively due to the production of surfactant molecules. On the contrary, KS 293 did not show any significant adaptation mechanism in its capacity to grow on the different substrates, with analogous physiological responses to pyruvate and C12. This relevant difference between the strains was reflected in a differential expression of genes related to carbon utilization pathways: despite comparable growth efficiency on C12, SK2 mostly overexpressed genes related to fatty acids biosynthesis and elongation, while KS 293 overexpressed genes related to β-oxidation, Krebs cycle and oxidative phosphorylation. The capacity of the strains to utilize carbon sources alternative to hydrocarbons would sustain them to permanently colonize pristine marine environments, while their differential transcriptomic responses to hydrocarbons could make them a potential functionally redundant and plastic reservoir of oil-degrading players, able to activate in the case of oil-spill occurrence, sustaining natural attenuation and biostimulation based clean-up processes.

## Author contributions

NB, SB, and DD conceived, supervised and funded the project; MB and AS designed the experimental plan; MB, AS, FM, RD, and IB performed the experiments; MB, AS, NB, and SB discussed the results; MB and SB wrote the manuscript; all revised the manuscript.

### Conflict of interest statement

The authors declare that the research was conducted in the absence of any commercial or financial relationships that could be construed as a potential conflict of interest. The reviewer HFS and handling Editor declared their shared affiliation, and the handling Editor states that the process nevertheless met the standards of a fair and objective review.
