# Supplementary material for: Hydrocarbonoclastic Alcanivorax Isolates Exhibit Different Physiological and Expression Responses to n-dodecane
Source: Front Microbiol. 2016 Dec 21;7:2056. doi: 10.3389/fmicb.2016.02056 (PMC5174103; doi:10.3389/fmicb.2016.02056)
Supplement: Supplementary file 2 [file Presentation1.PPTX]

## Slide 1
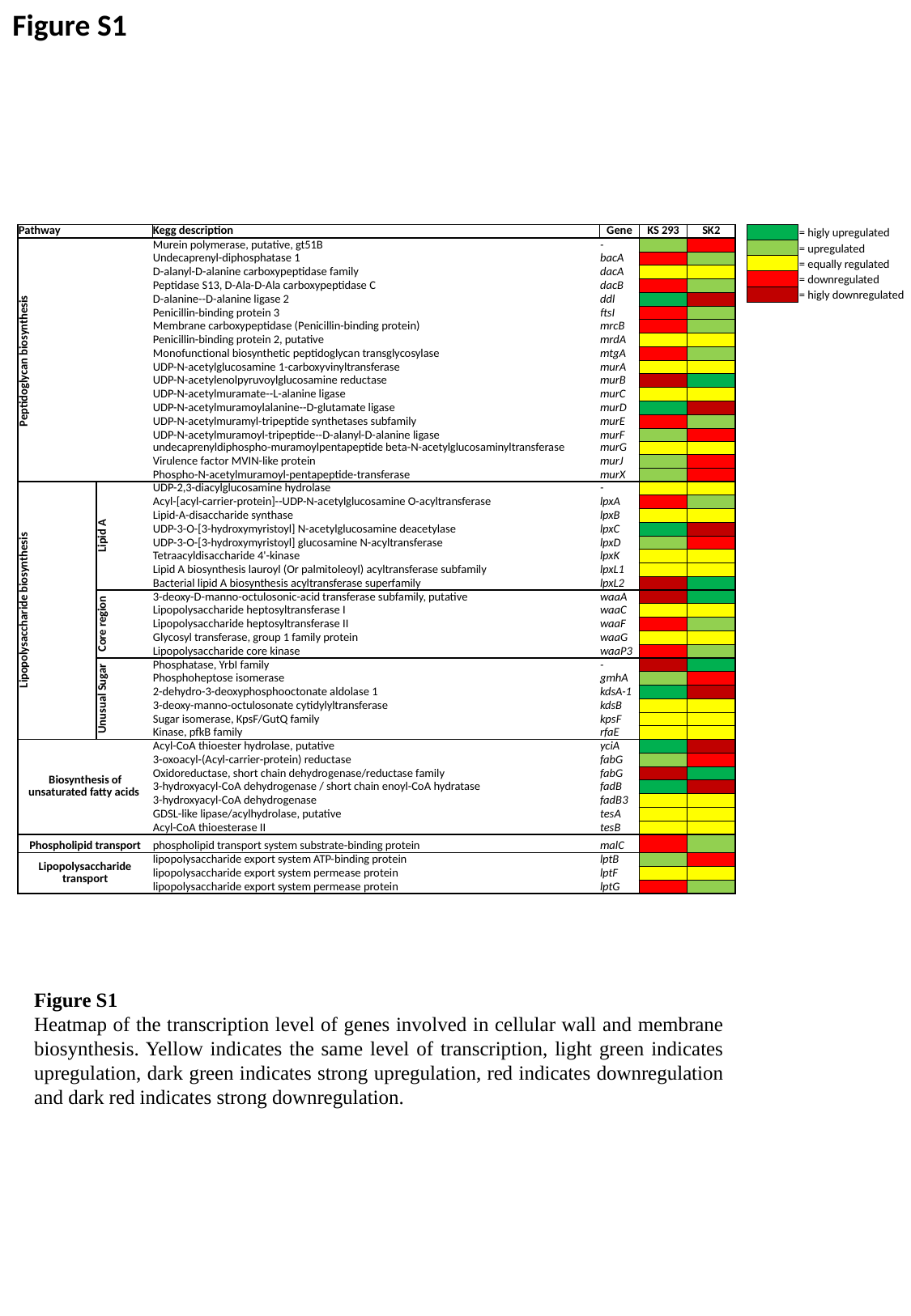

Figure S1
| Pathway | | Kegg description | Gene | KS 293 | SK2 |
| --- | --- | --- | --- | --- | --- |
| Peptidoglycan biosynthesis | | Murein polymerase, putative, gt51B | - | | |
| | | Undecaprenyl-diphosphatase 1 | bacA | | |
| | | D-alanyl-D-alanine carboxypeptidase family | dacA | | |
| | | Peptidase S13, D-Ala-D-Ala carboxypeptidase C | dacB | | |
| | | D-alanine--D-alanine ligase 2 | ddl | | |
| | | Penicillin-binding protein 3 | ftsI | | |
| | | Membrane carboxypeptidase (Penicillin-binding protein) | mrcB | | |
| | | Penicillin-binding protein 2, putative | mrdA | | |
| | | Monofunctional biosynthetic peptidoglycan transglycosylase | mtgA | | |
| | | UDP-N-acetylglucosamine 1-carboxyvinyltransferase | murA | | |
| | | UDP-N-acetylenolpyruvoylglucosamine reductase | murB | | |
| | | UDP-N-acetylmuramate--L-alanine ligase | murC | | |
| | | UDP-N-acetylmuramoylalanine--D-glutamate ligase | murD | | |
| | | UDP-N-acetylmuramyl-tripeptide synthetases subfamily | murE | | |
| | | UDP-N-acetylmuramoyl-tripeptide--D-alanyl-D-alanine ligase | murF | | |
| | | undecaprenyldiphospho-muramoylpentapeptide beta-N-acetylglucosaminyltransferase | murG | | |
| | | Virulence factor MVIN-like protein | murJ | | |
| | | Phospho-N-acetylmuramoyl-pentapeptide-transferase | murX | | |
| Lipopolysaccharide biosynthesis | Lipid A | UDP-2,3-diacylglucosamine hydrolase | - | | |
| | | Acyl-[acyl-carrier-protein]--UDP-N-acetylglucosamine O-acyltransferase | lpxA | | |
| | | Lipid-A-disaccharide synthase | lpxB | | |
| | | UDP-3-O-[3-hydroxymyristoyl] N-acetylglucosamine deacetylase | lpxC | | |
| | | UDP-3-O-[3-hydroxymyristoyl] glucosamine N-acyltransferase | lpxD | | |
| | | Tetraacyldisaccharide 4'-kinase | lpxK | | |
| | | Lipid A biosynthesis lauroyl (Or palmitoleoyl) acyltransferase subfamily | lpxL1 | | |
| | | Bacterial lipid A biosynthesis acyltransferase superfamily | lpxL2 | | |
| | Core region | 3-deoxy-D-manno-octulosonic-acid transferase subfamily, putative | waaA | | |
| | | Lipopolysaccharide heptosyltransferase I | waaC | | |
| | | Lipopolysaccharide heptosyltransferase II | waaF | | |
| | | Glycosyl transferase, group 1 family protein | waaG | | |
| | | Lipopolysaccharide core kinase | waaP3 | | |
| | Unusual Sugar | Phosphatase, YrbI family | - | | |
| | | Phosphoheptose isomerase | gmhA | | |
| | | 2-dehydro-3-deoxyphosphooctonate aldolase 1 | kdsA-1 | | |
| | | 3-deoxy-manno-octulosonate cytidylyltransferase | kdsB | | |
| | | Sugar isomerase, KpsF/GutQ family | kpsF | | |
| | | Kinase, pfkB family | rfaE | | |
| Biosynthesis of unsaturated fatty acids | | Acyl-CoA thioester hydrolase, putative | yciA | | |
| | | 3-oxoacyl-(Acyl-carrier-protein) reductase | fabG | | |
| | | Oxidoreductase, short chain dehydrogenase/reductase family | fabG | | |
| | | 3-hydroxyacyl-CoA dehydrogenase / short chain enoyl-CoA hydratase | fadB | | |
| | | 3-hydroxyacyl-CoA dehydrogenase | fadB3 | | |
| | | GDSL-like lipase/acylhydrolase, putative | tesA | | |
| | | Acyl-CoA thioesterase II | tesB | | |
| Phospholipid transport | | phospholipid transport system substrate-binding protein | malC | | |
| Lipopolysaccharide transport | | lipopolysaccharide export system ATP-binding protein | lptB | | |
| | | lipopolysaccharide export system permease protein | lptF | | |
| | | lipopolysaccharide export system permease protein | lptG | | |
| | = higly upregulated |
| --- | --- |
| | = upregulated |
| | = equally regulated |
| | = downregulated |
| | = higly downregulated |
Figure S1
Heatmap of the transcription level of genes involved in cellular wall and membrane biosynthesis. Yellow indicates the same level of transcription, light green indicates upregulation, dark green indicates strong upregulation, red indicates downregulation and dark red indicates strong downregulation.

## Slide 2
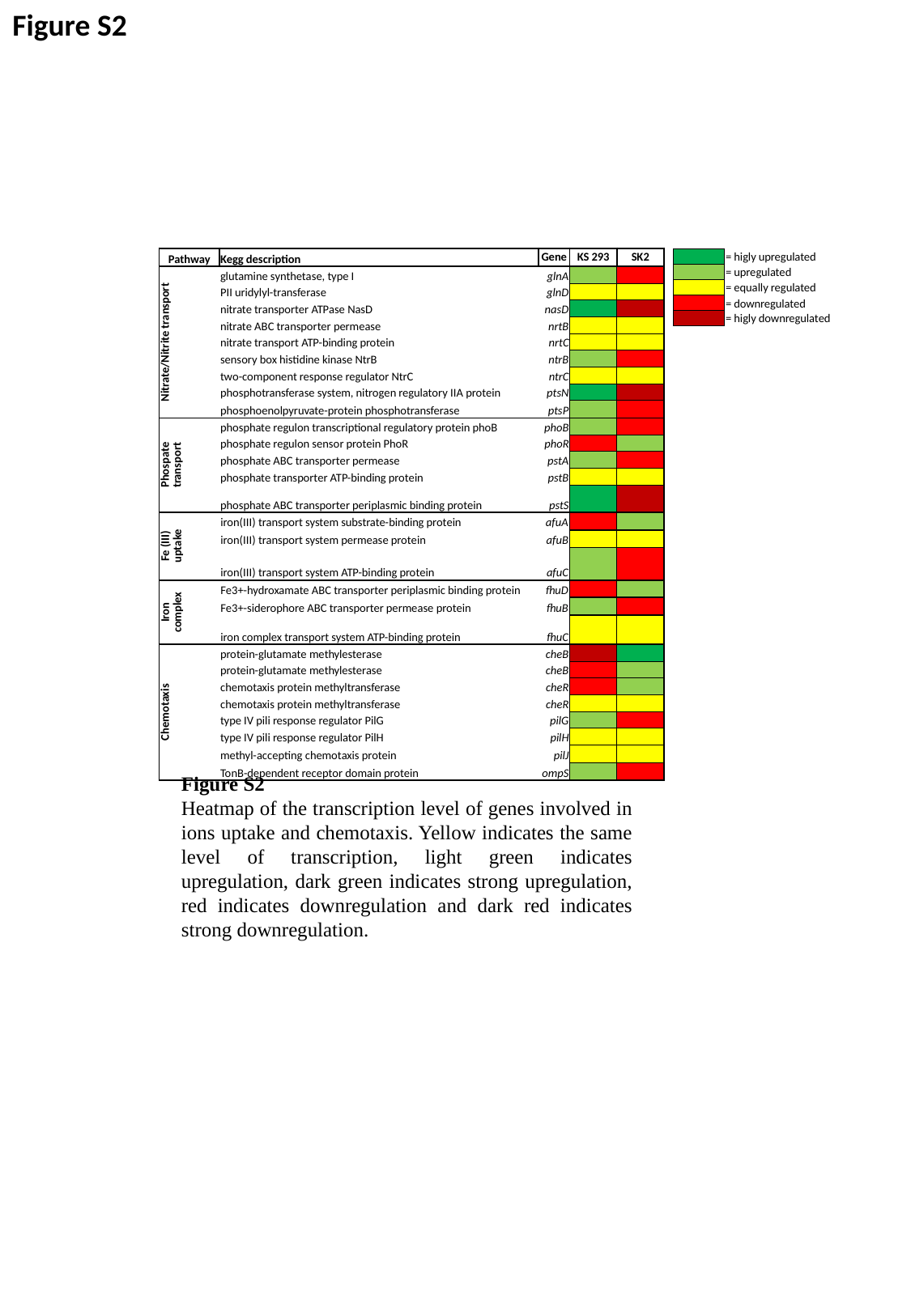

Figure S2
| Pathway | Kegg description | Gene | KS 293 | SK2 |
| --- | --- | --- | --- | --- |
| Nitrate/Nitrite transport | glutamine synthetase, type I | glnA | | |
| | PII uridylyl-transferase | glnD | | |
| | nitrate transporter ATPase NasD | nasD | | |
| | nitrate ABC transporter permease | nrtB | | |
| | nitrate transport ATP-binding protein | nrtC | | |
| | sensory box histidine kinase NtrB | ntrB | | |
| | two-component response regulator NtrC | ntrC | | |
| | phosphotransferase system, nitrogen regulatory IIA protein | ptsN | | |
| | phosphoenolpyruvate-protein phosphotransferase | ptsP | | |
| Phospate transport | phosphate regulon transcriptional regulatory protein phoB | phoB | | |
| | phosphate regulon sensor protein PhoR | phoR | | |
| | phosphate ABC transporter permease | pstA | | |
| | phosphate transporter ATP-binding protein | pstB | | |
| | phosphate ABC transporter periplasmic binding protein | pstS | | |
| Fe (III) uptake | iron(III) transport system substrate-binding protein | afuA | | |
| | iron(III) transport system permease protein | afuB | | |
| | iron(III) transport system ATP-binding protein | afuC | | |
| Iron complex | Fe3+-hydroxamate ABC transporter periplasmic binding protein | fhuD | | |
| | Fe3+-siderophore ABC transporter permease protein | fhuB | | |
| | iron complex transport system ATP-binding protein | fhuC | | |
| Chemotaxis | protein-glutamate methylesterase | cheB | | |
| | protein-glutamate methylesterase | cheB | | |
| | chemotaxis protein methyltransferase | cheR | | |
| | chemotaxis protein methyltransferase | cheR | | |
| | type IV pili response regulator PilG | pilG | | |
| | type IV pili response regulator PilH | pilH | | |
| | methyl-accepting chemotaxis protein | pilJ | | |
| | TonB-dependent receptor domain protein | ompS | | |
| | = higly upregulated |
| --- | --- |
| | = upregulated |
| | = equally regulated |
| | = downregulated |
| | = higly downregulated |
Figure S2
Heatmap of the transcription level of genes involved in ions uptake and chemotaxis. Yellow indicates the same level of transcription, light green indicates upregulation, dark green indicates strong upregulation, red indicates downregulation and dark red indicates strong downregulation.
